# Establishing a novel model of malignant airway stenosis in rabbit

**DOI:** 10.3389/fonc.2022.959309

**Published:** 2022-08-18

**Authors:** Xiaoxiao Lin, Liqin Zhou, Wanting Zhou, Yuping Li, Xuru Jin, Min Ye, Chengshui Chen

**Affiliations:** ^1^ Department of Pulmonary and Critical Care Medicine, First Affiliated Hospital of Wenzhou Medical University, Wenzhou, China; ^2^ The Quzhou Affiliated Hospital of Wenzhou Medical University, Quzhou People’s Hospital, Quzhou, China; ^3^ Key Laboratory of Interventional Pulmonology of Zhejiang Province, First Affiliated Hospital of Wenzhou Medical University, Wenzhou, China

**Keywords:** malignant airway stenosis, animal model, rabbit, bronchoscopy, VX2

## Abstract

**Background:**

Malignant central airway stenosis is a life-threatening condition. However, treatment of malignant airway stenosis remains challenging. There is currently a severe lack of an excellent animal model of malignant airway stenosis to facilitate treatment approaches. This is the first study to establish a rabbit model of malignant airway stenosis for bronchoscopic interventional studies.

**Materials and methods:**

New Zealand White rabbits were used in this study, randomly divided into group A (18 rabbits) and group B (6 rabbits). A VX2 fragment suspension was injected into the submucosal layer of rabbits’ airway by bronchoscopy. Bronchoscopic examinations were performed once a week after VX2 tumor implantation to observe tumor growth and the degree of airway stenosis. Randomly, three rabbits were generally dissected after a weekly bronchoscopic examination in group A. The rabbits that reached grade III airway stenosis underwent stent implantation in group B.

**Results:**

A total of 24 rabbits were successfully implanted with the VX2 fragment suspension in the airway without significant adverse events, and the success rate of the tumor growth was 100%. The degree of airway stenosis reaching grade III took 2 to 3 weeks after implantation of the VX2 tumor. The median survival time of rabbit models without stent implantation and rabbits with stent implantation was 32.5 and 32.0 days, respectively.

**Conclusions:**

The implanting method is safe and effective for the establishment of a rabbit model of malignant airway stenosis. When the tumor grows to 2 to 3 weeks, the rabbit model is available for stent implantation. We recommend the models for more preclinical animal studies on bronchoscopic interventional treatments.

## Introduction

Malignant central airway stenosis is a life-threatening condition caused by malignant tumors, most commonly occurring in locally advanced lung cancer (1, 2). For patients ineligible for surgery due to poor general condition or an advanced tumor stage, bronchoscopic intervention is a useful treatment option, including photodynamic therapy, tumor ablation, cryotherapy, and airway stenting (3). However, treatment of malignant airway stenosis remains challenging and is variable among clinicians and institutions (4, 5). Besides other techniques, stent-related technologies continue to overcome the current complications of stents, such as migration, infection, and granulation tissue formation (6). Recently, novel stents, new laser, and spray cryotherapy with a novel multimodal approach have been gradually designed to solve malignant airway stenosis (7–11). However, the ideal bronchoscopic technique does not currently exist, and studies on new techniques are necessary and significant.

Although many of these technical changes can be evaluated directly in the patient, clinical investigation often needs preliminary support from animal studies. However, there is currently a severe lack of an excellent animal model of malignant airway stenosis to facilitate treatment-related studies.

The VX2 tumor, a squamous cell cancer model, which was firstly proposed by Shope and Hurst (12) in 1933, has been implanted in many sites of rabbits, including the liver, kidney, lung, esophagus, and muscle (13–15). Although the rabbits belong to moderate-to-large-sized models and have been used to study benign airway stenosis in stent-related research (16, 17), there have been no reports on establishing a rabbit model with malignant airway stenosis.

This is the first study that aims to develop a rabbit model of malignant airway stenosis in which we describe the use of submucosal VX2 fragments in the tracheal wall and to investigate the safety and efficiency of the method, as well as the characteristics of the airway stenosis model. Furthermore, we observed the feasibility of stent implantation in the novel model.

## Materials and methods

### Animal and tumor

Male or female New Zealand White rabbits (Kelian Rabbit Professional Cooperative, Hangzhou, China) weighing between 2.5 and 3.5 kg were used in this study. The VX2 tumor previously stored at -80°C was obtained from the Surgery Laboratory in the First Affiliated Hospital of Wenzhou Medical University. All experimental procedures were approved by the Laboratory Animal Center of Wenzhou Medical University (ID: wydw2021-0289) and were performed in accordance with the National Institute of Health’s Guide for the Care and Use of Laboratory Animals.

### Tumor propagation and implantation preparation

Four rabbits with hind-limb tumors were used to propagate and maintain VX2 tumors. Approximately 0.2 ml of saline with VX2 tumor tissue fragments, which could pass through a 1-ml syringe connecting with an 18-gauge needle, was injected deeply into the hind limb of gluteal muscles of rabbits. After 2~3 weeks, the animals were executed and the hind-limb tumors were processed. All VX2 tumors were cleaned from the surrounding normal tissue, and necrotic portions of the tumors were removed.

**Figure 1:**
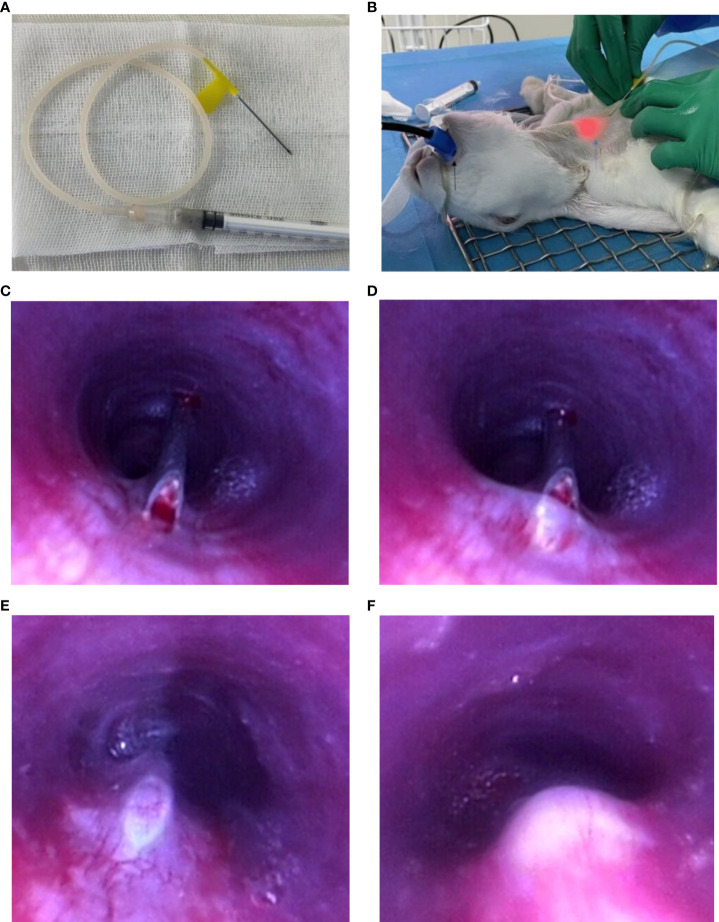
Endoscopic implantation of VX2 fragment suspension. Preparation before implantation: **(A)** VX2 fragment suspension in a 1-ml syringe connecting with a 20-gauge transfusion needle. **(B)** The black arrow points to the medical tooth pad; blue arrow points to the light source of the bronchoscope; Procedure of endoscopic implantation: **(C–E)** The 20-guage needle entering into the submucosal layer. **(F)** Injection of VX2 fragment suspension.

The collected tumors were cut into small pieces approximately 0.5 mm in diameter, which could pass through the 20-gauge needle, and preserved in saline for fragment implantation. Approximately 0.6 ml tumor fragment suspension was placed in a 1-ml syringe connecting with a 20-gauge transfusion needle (**Figure 1A**).

### Endoscopic implantation of VX2 fragment suspension

A total of 24 rabbits were included in the process and randomly divided into group A and group B. Group A (18 rabbits) was used to observe the characteristics of the airway stenosis model, and group B (6 rabbits) was implanted with a metal stent when malignant stenosis occurred in the rabbit airway.

Each rabbit was firstly anesthetized by injection with 3% pentobarbital sodium (1 ml/kg) *via* the ear vein and then secured in supine position. Its anterior neck was exposed, and then preoperative hair removal was prepared. A medical tooth pad was fixed in the oral cavity of the rabbit. An ordinary bronchoscope with an outer diameter of 4.9 mm (UE Medical Corporation, Zhejiang, China) was inserted into the airway from the tooth pad, and 1 ml of lidocaine (2%) was injected into the vocal area to reduce the intense cough reaction of the rabbit.

Then the anterior neck between two tracheal cartilages was punctured with the 20-gauge transfusion needle in the guidance of a light source of the bronchoscope (**Figure 1B**). The puncture site was approximately located 1 cm away from the upper part of the manubrium sternum. After inserting through the skin, subcutaneous tissue, and anterior tracheal wall to the airway, a puncture was made using the needle across the mucosa into the submucosal layer in the tracheal membrane. When the whole inclined surface of the needle enters into the submucosal layer, which could be clearly seen by a bronchoscope, approximately 0.15 ml of VX2 fragment suspension was then injected into the submucosal layer of the rabbit’s airway. Finally, the needle was rapidly evacuated from the airway. The operational process is vividly shown in **Figures 1C–F** and **Video 1**. The implantation site was approximately 5 cm away from the tracheal carina, which was the equivalent of 3 cm away from the glottis of the rabbit. During the procedure, a 50-ml syringe was put into the working channel of the bronchoscope to artificially provide oxygen to the rabbit. The complications of the procedure, such as apnea, death, and massive hemorrhage, were assessed and accessed immediately after the procedure. After implantation, the rabbits were intramuscularly injected with 200,000 U penicillin at once.

### Endoscopic follow-up

Bronchoscopic examinations were performed once a week after VX2 tumor implantation to observe tumor growth and the degree of airway stenosis. The anesthesia procedure was the same as that described before. Referring to the published classification system of airway stenosis (18), four grades were used to evaluate the airway stenosis under endoscopy: grade I stenosis less than or equal to a 25% decrease in the lumen cross-sectional area; grade II stenosis more than 25% but less than or equal to 50%; grade III stenosis more than 50% but less than or equal to 75%; and grade IV stenosis more than 75%.

### Survival time and necropsy

In group A, randomly three rabbits were generally dissected after a weekly bronchoscopic examination. In other words, every three rabbits were executed in the first, second, third, and fourth weeks after tumor implantation. The remaining six rabbits, without stent implantation, were observed until they had inability to survive. A humane endpoint was used in this study, at which the animal models would be humanely sacrificed when they had significant symptoms of dyspnea and wheeze and then their health was so weak that they could not stand and eat food.

The rabbits were injected with 3% pentobarbital sodium (1 ml/kg) *via* the ear vein and then injected with 20 ml of air. General necropsy examinations involved the organs of the tracheal wall, esophagus, lung, liver, and kidney, to clarify tumor invasion and metastasis. The length and width and height of the VX2 tumor in the trachea were measured by a vernier caliper. All the tumors were evaluated histologically. Hematoxylin–eosin (HE)-stained histopathology is the gold standard for the diagnosis of carcinoma.

### Stent implantation

In group B, a self-expandable metal stent (8 mm in diameter and 30 mm in length) was implanted when the degree of airway stenosis in the rabbit reached grade III. Firstly, the distance from the distal end of the airway tumor to medical teeth was measured by bronchoscopy and a guidewire was inserted across the stenosis into the main bronchus. Then the delivery device was gently passed over the guidewire and the stent was released according to the measured distance before. After withdrawing the delivery device, ideal positioning of the stent was assessed and adjusted endoscopically. Finally, bronchoscopic examinations were performed every week to observe the stent-related complications and airway restenosis.

## Results

### Safety of experimental procedure

All 24 rabbits tolerated the bronchoscopy procedure without clinically significant adverse events. When implanting VX2 fragment suspension, no apnea or massive hemorrhage, or even death, occurred (**Table 1**). It would only take 60 s from bronchoscope inserting into the airway to bronchoscope withdraw.

**Table 1:** The observational data in the study.

Observation	Number	Percent
Implanting VX2 fragment suspension		
Success	24/24	100%
Hemorrhage	0/24	0%
Apnea	0/24	0%
Death	0/24	0%
Success rate of tumor growth	24/24	100%
Success rate of stenosis model in group A		
First week	8/18	44.4%
Second week	13/15	86.7%
Third week	12/12	100%
Implanting stent in group B		
Success rate	6/6	100%
Complication		
Airway restenosis	4/6	66.7%
Airway cancerous fistula	1/6	16.7%
Granulation tissue formation	1/6	16.7%

### Success rate of tumor growth

In the first week after tumor implantation, small nodules attached to the outer wall of the trachea in all living rabbits. All rabbits were confirmed to have tumor growth in their airway by dissection and then histopathology. The success rate of tumor growth in the rabbit airway was 100% (**Table 1**).

### Tracheal tumor feature

**Figure 2:**
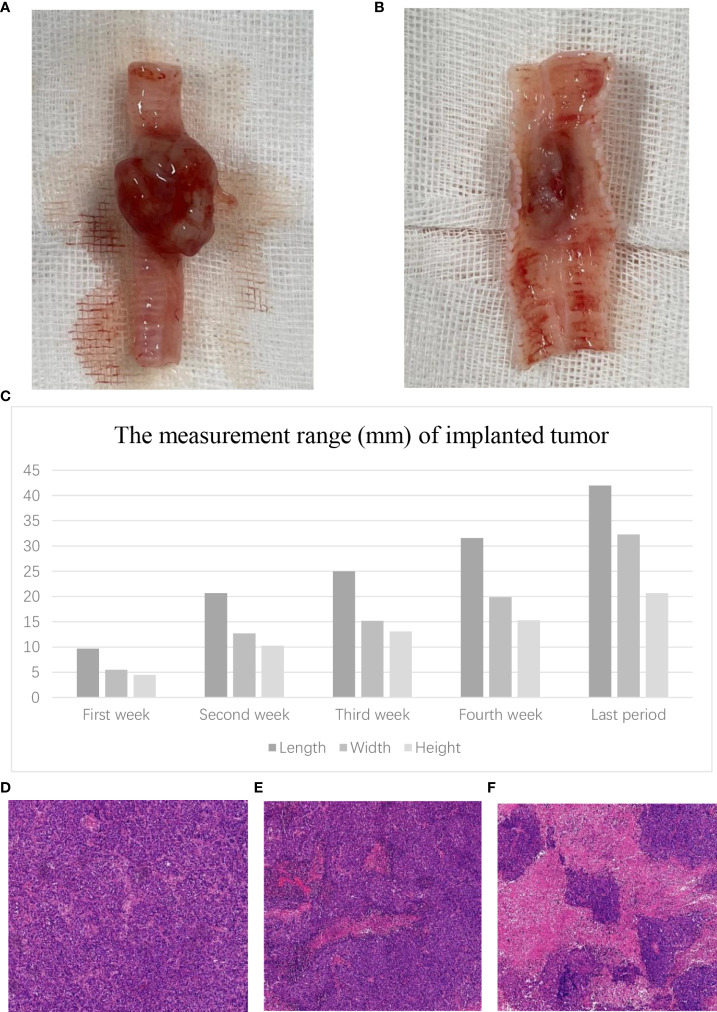
Tumor growth in the airway. **(A)** Macroscopic external view of the tracheal tumor. **(B)** Macroscopic internal view of the tracheal tumor. **(C)** The measurement range of the VX2 tumor in different periods. Microscopic view of the tracheal tumor. **(D)** Tumor cells grew vigorously. **(E)** Tumor showed slightly necrotic portions. **(F)** Tumor showed obviously necrotic portions.

In group A, the macroscopic external and internal views of the tracheal tumor are as shown in **Figures 2A, B**. The mean length of the airway tumor is 9.7 mm in the first week; 20.7 mm in the second week; 25.0 mm in the third week; 31.6 mm in the fourth week; and 42 mm in the last rabbits. Furthermore, the mean width (height) is respectively 5.5 (4.5) mm, 12.7 (10.3) mm, 15.2 (13.1) mm, 19.9 (15.3) mm, and 32.3 (20.5) mm in different periods of rabbits (**Figure 2C**).

In histopathological examination, the tumor cells grew vigorously in the first 3 weeks. Moreover, some tumors (22.2%, 2/9) showed slightly necrotic portions in the second and third weeks. However, the tumors showed obviously necrotic portions in the fourth week (**Figures 2D–F**).

### Success rate of establishing malignant airway stenosis

In group A, during the bronchoscopic follow-up after tumor implantation, non-appreciable airway stenosis, I grade stenosis, and grade II stenosis were observed in 56% (10/18), 39% (7/18), and 5% (1/18) rabbits in the first week, respectively.

In the second week, the percentages of non-appreciable stenosis, grade I stenosis, grade II stenosis, and grade III stenosis were 13% (2/15), 34% (5/15), 40% (6/15), and 13% (2/15), respectively. Moreover, in the third week, the percentages of grade II stenosis, grade III stenosis, and grade IV stenosis were 42% (5/12), 42% (5/12), and 16% (2/12), respectively. In the fourth weekly bronchoscopic examination, three rabbits (33%) showed grade III airway stenosis and six rabbits (67%) showed grade IV stenosis. The data are summarized in **Table 2**. Moreover, rabbits with grade IV airway stenosis had symptoms of breathing difficulties and weakness and suffered from massive mucilage secretion plugging in the distal airway.

**Table 2:** Degree of airway stenosis in different weeks after tumor implantation.

Rabbit, no. (%)	Non-appreciate	I grade	II grade	III grade	IV grade
First week, 18	10 (56%)	7 (39%)	1 (5%)		
Second week, 15	2 (13%)	5 (34%)	6 (40%)	2 (13%)	
Third week, 12			5 (42%)	5 (42%)	2 (16%)
Fourth week, 9				3 (33%)	6 (67%)

### Airway stenosis types

**Figure 3:**
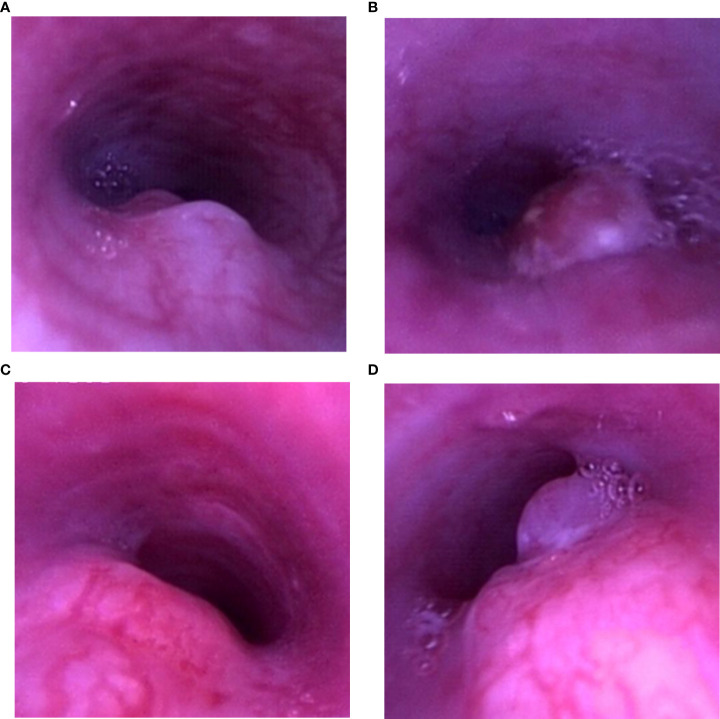
Airway stenosis types. **(A)** Tumor invasion of the airway wall. **(B)** Intraluminal tumor. **(C)** Extrinsic compression of the airway. **(D)** Combination growth.

The gross classical appearance of the tumors varied from small submucosal microbulge to larger mass in bronchoscopy. In rabbits with malignant airway stenosis in the study, different structural stenosis types including extrinsic compression of the airway, tumor invasion of the airway wall or intraluminal tumor, and a combination growth are shown in **Figure 3**.

### Tumor distant metastasis

**Figure 4:**
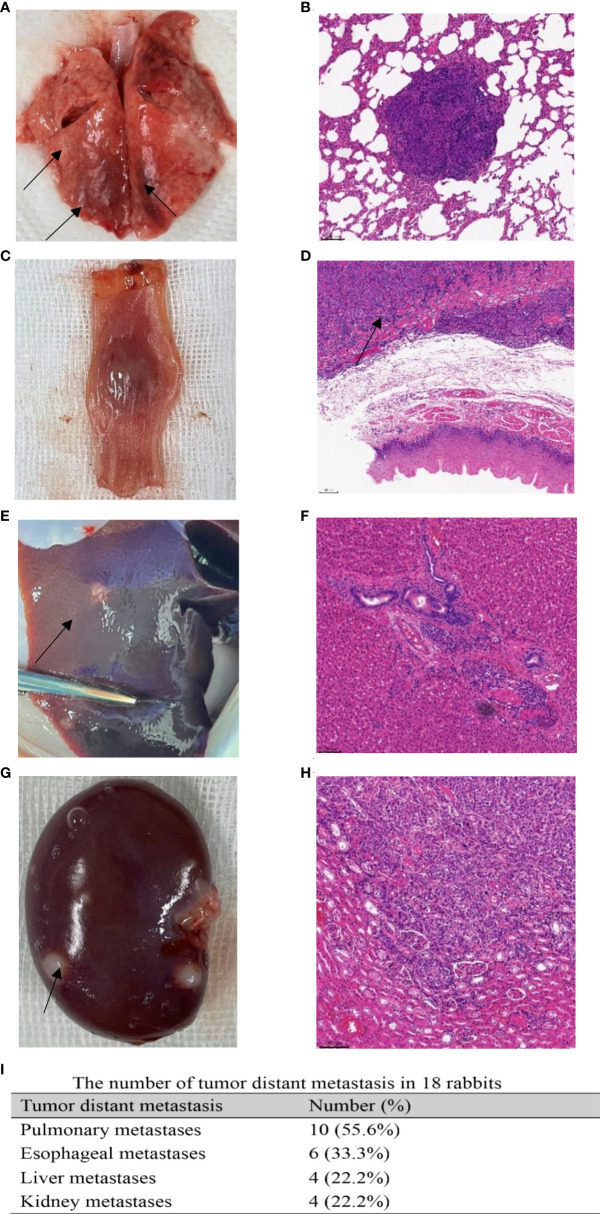
Tumor distant metastases. Macroscopic view of tumor metastases: **(A)** pulmonary metastasis (black arrow); **(C)** esophageal metastasis; **(E)** hepatic metastasis (black arrow); **(G)** renal metastasis (black arrow). Microscopic observation of tumor metastases: **(B)** pulmonary metastasis; **(D)** esophageal metastasis. The black arrow points to cancer cells infiltrating the esophageal muscular layer. **(F)** Hepatic metastasis. **(H)** Renal metastasis. **(I)** The number of tumor distant metastases in 18 rabbits.

The airway tumor did not metastasize to other organs of rabbits in the first and second weekly necropsy examinations. In the third week, one rabbit was observed to have diffuse pulmonary metastases. In the fourth week, three rabbits were verified with diffuse pulmonary metastases while one of them was attacked by metastatic esophageal carcinoma. The remaining rabbits with their survival time of more than 28 days all showed pulmonary metastases; one of them also had esophageal metastasis, and four of them presented with multiple metastatic organs including lung, esophagus, liver, and kidney. The manifestations of metastatic lesions and corresponding histopathology are demonstrated in **Figure 4**.

### Survival time of the rabbit models

The remaining six rabbits in group A suffered from respiratory failure. Before they received humane sacrifice, the bronchoscopic examination presented grade IV stenosis on the airway. The survival time of rabbits varied from 31 to 34 days, and the median survival time was 32.5 days after VX2 tumor implantation.

### Stent-related complications

**Figure 5:**
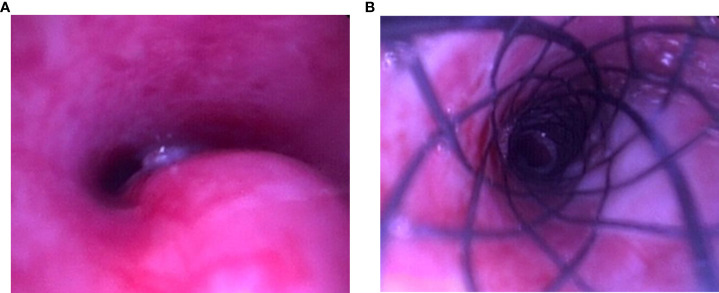
Stent implantation. **(A)** Malignant airway stenosis. **(B)** Airway opened.

In group B, the time of airway showing grade III stenosis was 3 weeks in four rabbits and 2 weeks in other rabbits. The metal stents were successfully implanted into the narrow airway of six rabbits. Moreover, the airway of all six rabbits recovered patency at once after stent implantation (**Figure 5**). The median survival time of relevant rabbits was 32.0 days after VX2 tumor implantation. Four rabbits died of severe airway restenosis and one died of airway cancerous fistula, and the remaining one rabbit suffered granulation tissue formation and mucus plugging. The observational data in the study are shown in **Table 2**.

## Discussion

In the current study, the bronchoscopic procedure was minimally invasive with a high success rate of implantation and establishment of a malignant airway stenosis model. The adequate follow-up data showed a degree of airway stenosis in different periods, different airway stenosis types, survival time, tumor growth, and metastases of rabbits after VX2 implantation, which indicated that the animal models were reliable for mimicking the disease of human malignant central airway stenosis. When the tumor grows to 2 to 3 weeks, the rabbit model is available for stent implantation.

Malignant central airway stenosis is a serious condition that causes the death of patients. Patients with malignant airway stenosis always have a poor prognosis because of respiratory obstructions and reduced quality of life. Interventional bronchoscopy, especially airway stenting, is an available treatment to palliation of symptoms. However, adequate animal studies are necessary to evaluate the efficacy and safety of bronchoscopy-related treatments. In a recent study, animals were not under disease conditions and were only used to assess the safety of a stent loaded with ^125^I seeds, due to the current lack of animal models of malignant airway stenosis (19). Therefore, a reliable animal model of malignant airway stenosis is sorely needed.

Small animal models, such as mice, are reasonable options for evaluating systemic therapy but have limitations for evaluating bronchoscopic technologies. Even though the airway structure of large animal models are more similar to humans, management of dogs or pigs is extremely difficult for researchers. However, New Zealand White rabbits are readily available and relatively inexpensive as models and are sufficiently large to allow the oral insertion of bronchoscope to their airway. Moreover, rabbits allow for research on bronchoscopic interventional treatments (20, 21). Prof. Li et al. implanted a novel stent into a rabbit airway to evaluate its function (17). Nakagishi et al. studied the application of photodynamic therapy for a benign airway stenosis rabbit model under bronchoscopy (22). All in all, the New Zealand White rabbit is a rational model to be adopted to perform the animal trial. According to our observations, the bronchoscope could smoothly pass through the airway of rabbits. Moreover, rabbits could tolerate the bronchoscopy procedure without severe complications. The rabbit model of malignant airway stenosis exhibits the characteristics of malignant stenosis and shows potential use for research on the efficiency of bronchoscopic treatments.

It has been reported that the success rate of tumor growth by the implantation of a VX2 fragment approximately 1 mm in diameter was higher than that by implantation of VX2 cells in the liver of rabbits (23, 24). The current data demonstrated that the method of implanting VX2 fragment suspension into the submucosal layer of the rabbit airway using a bronchoscope resulted in a 100% success in tumor growth.

It is a significant advantage on tumor implantation and then follow-up under bronchoscopic monitoring. The operational process could be clearly seen by bronchoscopy, and the condition of malignant airway stenosis was observed dynamically. Moreover, there was no apnea and death of rabbits during bronchoscopy.

Occlusion of >50% of the trachea in central malignant airway stenosis always leads to obvious clinical symptoms of patients and requires bronchoscopic interventional treatments. In the study, the degree of airway stenosis reaching grade III only took 2 to 3 weeks after implantation of a VX2 tumor. It signifies that the researchers can rapidly and conveniently establish a malignant stenosis model in rabbits, which is conducive to further studies on airway treatment. The supplementary experiment showed that a rabbit model with malignant airway stenosis could tolerate stenting implantation and then respiratory obstructions could be alleviated, although the bare metal stent did not obviously increase the survival time of rabbits because of tumor ingrowth and stent-related complications during the follow-up. These results indicate that it is safe and feasible to perform bronchoscopic intervention in our animal models. Moreover, the tumor tissue showed obviously necrotic portions after tumor growth at 4 weeks, which may be followed by an occurrence of an airway cancerous fistula. Therefore, we consider that the rabbit model is available for bronchoscopic interventional studies when the tumor grows at 2 to 3 weeks.

A limitation of the model we created, however, is that the types of malignant airway stenosis could not be controlled after VX2 implantation. In addition, we just chose one dose (0.15 ml) of VX2 tissue suspension. Future studies are needed to observe the relationship between stenosis types and the amount of VX2 suspension.

In conclusion, the implanting method described above is safe and effective for the establishment of a rabbit model of malignant airway stenosis to mimic the progression of this human disease. When the tumor grows at 2 to 3 weeks, the rabbit model is available for stent implantation. We recommend the model for more preclinical animal studies on bronchoscopic interventional treatments.

## Data availability statement

The original contributions presented in the study are included in the article/**Supplementary Material**. Further inquiries can be directed to the corresponding author.

## Ethics statement

The animal study was reviewed and approved by Laboratory Animal Center of Wenzhou Medical University (ID: wydw2021-0289).

## Author contributions

XL was a major contributor in writing the manuscript. CC and YL designed the study. MY and XJ analyzed the data. LZ and WZ performed the study. All authors contributed to the manuscript revision and read and approved the submitted version.

## Funding

The study was supported by grants from the Key Laboratory of Interventional Pulmonology of Zhejiang Province (2019E10014) and the Zhejiang Provincial Key Research and Development Program (2020C03067).

## Conflict of interest

The authors declare that the research was conducted in the absence of any commercial or financial relationships that could be construed as a potential conflict of interest.

## Publisher’s note

All claims expressed in this article are solely those of the authors and do not necessarily represent those of their affiliated organizations, or those of the publisher, the editors and the reviewers. Any product that may be evaluated in this article, or claim that may be made by its manufacturer, is not guaranteed or endorsed by the publisher.

## Supplementary material

Supplementary Video 1 Procedure of endoscopic implantation.
